# Evaluation of *Rhinella marina* Extracts on the Effector Functions of Human Neutrophils

**DOI:** 10.3390/biology15191770

**Published:** 2026-10-04

**Authors:** Lucinéia Reuse Albiero, Valfran da Silva Lima, Cleni Mara Marzocchi-Machado, Luiz Fernando Fortunato Tucci, Antônio Carlos Alves De Sousa, Domingos de Jesus Rodrigues, Lucas Castro Fernandes Pimenta, Marcos Penhacek, Marcilei Juvenal da Conceição Horn, Jessyca Hathenher Gomes, Valéria Dornelles Gindri Sinhorin, Adilson Paulo Sinhorin

**Affiliations:** 1 Postgraduate Program in Biotechnology and Biodiversity of the Pró Centro-Oeste Network, Federal University of Mato Grosso, Sinop 78557-267, MT, Brazil; 2 Institute of Health Sciences, Federal University of Mato Grosso, Sinop 78557-267, MT, Brazil; 3 Department of Clinical Analyses, Toxicology and Food Sciences, School of Pharmaceutical Sciences of Ribeirão Preto, University of São Paulo, Ribeirão Preto 14040-903, SP, Brazil; 4 Postgraduate Program in Ecology and Biodiversity Conservation, Federal University of Mato Grosso, Cuiabá 78060-900, MT, Brazil; 5 Secretariat of Science, Technology and Innovation of the State of Mato Grosso, Cuiabá 78020-450, MT, Brazil; 6 Postgraduate Program in Chemistry, Federal University of Mato Grosso, Cuiabá 78060-900, MT, Brazil

**Keywords:** *Rhinella marina*, neutrophils, immunomodulation

## Abstract

Neutrophils play a central role in the innate immune response. However, in inflammatory and autoimmune diseases, the exacerbated activation of these cells can result in excessive release of reactive oxygen species (ROS), proteases, and inflammatory mediators, contributing to tissue damage. In this context, natural compounds derived from amphibian venoms have attracted interest due to their pharmacological and immunomodulatory potential. This study aimed to evaluate in vitro the effects of the crude methanolic extract (CME) of *Rhinella marina* on the effector functions of human neutrophils. For this purpose, peripheral blood neutrophils from healthy individuals were treated with CME (0.5, 5, and 50 µg/mL) or untreated controls. Cytotoxicity, total ROS production, superoxide anion production, myeloperoxidase (MPO) activity, elastase degranulation, NET (Neutrophil Extracellular Trap) formation, and parameters related to cellular redox status, including antioxidant enzymes and markers of oxidative damage, were evaluated. CME showed concentration-dependent cytotoxicity. There were no changes in ROS production by luminol-dependent chemiluminescence after stimulation with myristoylphorbol acetate (PMA). However, it reduced superoxide anion production in neutrophils in both stimuli in the NBT assay. CME did not significantly modulate MPO activity or NET formation, but it reduced elastase release, suggesting modulation of neutrophil degranulation. Additionally, it increased glutathione-S-transferase (GST) activity and reduced glutathione (GSH) levels. It can be concluded that CME partially modulated neutrophil effector functions, such as superoxide anion production and degranulation, in addition to exhibiting partial protective effects on cellular redox state parameters. These findings suggest the extract’s immunomodulatory potential and reinforce the need for further studies to clarify its mechanisms of action and possible applicability in neutrophil-mediated inflammatory conditions.

## Simple Summary

This study evaluated the immunomodulatory effects of the crude methanolic extract (CME) of *Rhinella marina* on the effector functions of human neutrophils in vitro. The results demonstrated concentration-dependent cytotoxicity and selective modulation of neutrophil activity. Although no significant changes were observed in total reactive oxygen species production, myeloperoxidase activity, or NET formation, CME reduced superoxide anion production and elastase release, indicating a possible regulatory action on oxidative metabolism and degranulation. Furthermore, the extract promoted partial protective effects on the cellular redox state, increasing glutathione-S-transferase activity and reduced glutathione levels. The study’s strength lies in its comprehensive analysis of different neutrophil functions.

## 1. Introduction

Natural products represent one of the main sources for the development of new drugs, standing out as important reservoirs of bioactive molecules with therapeutic potential for different diseases, including inflammatory and autoimmune diseases [1]. In this context, the search for compounds capable of modulating exacerbated inflammatory responses remains a relevant strategy, especially given the therapeutic limitations and adverse effects associated with conventional treatments. Among the natural sources of bioactive compounds, animal poisons have aroused increasing scientific interest due to the wide diversity of biologically active molecules present in their composition, many of which have relevant pharmacological properties, including antimicrobial, antioxidant, anti-inflammatory, and immunomodulatory activities [2,3].

Brazilian biodiversity harbors a wide variety of anuran amphibians capable of producing biologically active cutaneous secretions [4]. Among these species, *Rhinella marina* (*R. marina*), an amphibian belonging to the Bufonidae family, widely distributed in the tropical regions of the Americas, stands out [5]. The secretions produced by the parotoid and cutaneous glands of this species constitute a complex chemical defense system composed of different bioactive metabolites, including bufadienolides, alkaloids, biogenic amines, peptides, and proteins [6,7,8]. Bufadienolides represent the main bioactive constituents of *R. marina* poison. Among them, marinobufagin, telocinobufagin, bufalin, and resibufogenin stand out as steroidal compounds capable of modulating physiological processes [2,9]. These metabolites have aroused increasing interest due to their cardiotonic, antimicrobial, antioxidant, antiplasmodial, antimutagenic, and antitumor properties [3,9,10]. In Brazil, the high biodiversity of amphibians, particularly in the Amazon, favors the biotechnological exploration of these secretions, increasing interest in bioprospecting for compounds with therapeutic potential [11,12,13]. Among the genera of interest, *Rhinella* stands out, whose secretions present significant molecular diversity and pharmacological potential that are still only partially explored [2,9].

In parallel, neutrophils constitute the most abundant leukocytes in the bloodstream and represent one of the main cellular components of innate immunity, being rapidly recruited to sites of infection or tissue injury [14]. These cells perform essential functions in host defense through effector mechanisms such as phagocytosis, production of reactive oxygen species (ROS), release of the microbicidal content of their granules, and formation of neutrophil extracellular traps (NETs), promoting the elimination of bacteria, fungi, and protozoa [15,16,17]. However, exacerbated neutrophil activation has been associated with the pathogenesis of several inflammatory and autoimmune diseases, contributing to the amplification of the inflammatory response and tissue damage [18]. In diseases such as rheumatoid arthritis, neutrophils from peripheral blood show increased ROS production, greater susceptibility to degranulation, and an exacerbated immune complex response when compared to neutrophils from healthy individuals [19]. Excessive ROS production is directly related to the establishment of oxidative stress, a condition characterized by an imbalance between the generation of reactive species and the ability of antioxidant systems to neutralize them, favoring damage to lipids, proteins, and nucleic acids and contributing to the perpetuation of chronic inflammation [20].

Although these mechanisms are fundamental to host defense, the excessive and nonspecific release of cytotoxic mediators can significantly contribute to the perpetuation of inflammation and damage to adjacent tissues [21]. In this scenario, modulating the effector functions of neutrophils emerges as a promising therapeutic strategy, aiming to reduce inflammatory damage without completely compromising the antimicrobial capacity of these cells [22].

Consequently, natural compounds with immunomodulatory properties have been investigated as potential complementary or adjuvant therapies for neutrophil-mediated inflammatory diseases [19,23]. Although studies demonstrate that secretions from amphibians of the Bufonidae family have relevant biological properties, including anti-inflammatory and immunomodulatory activity, information on the effects of *R. marina* poison on effector functions of human neutrophils is still limited.

Therefore, it becomes relevant to investigate the immunomodulatory potential of these secretions as an initial step towards identifying new bioactive compounds with therapeutic applications. Thus, the objective of this work was to evaluate, in vitro, the antioxidant and anti-inflammatory effects of the *R. marina* toad venom extract on the effector functions of peripheral blood neutrophils from healthy individuals.

## 2. Materials and Methods

### 2.1. Preparation of R. marina Poison Extract

Frogs of the species *R. marina* were captured and identified by the herpetologist team from ABAM-UFMT. The poison was obtained through manual compression of the parotoid gland, and the animals were then returned to nature (collection permit D. J. Rodrigues—IBAMA, SISBIO: number 30034-1).

*R. marina* poison extracts were prepared at the Phytochemistry Laboratory of the Federal University of Mato Grosso, Sinop University Campus, according to the methodology described by Pelissari et al. [3], obtained by extraction with methanol solvent at a ratio of 5 mg of crude poison per mL of methanol. The extraction procedure involved prior grinding of the poison, weighing on an analytical balance, dilution, maceration under ultrasound for 1 h, and filtration through analytical filter paper. After obtaining the extract, the solvent was removed under reduced pressure until constant weight was reached, yielding the CME. The extracts were solubilized in pure dimethyl sulfoxide (DMSO) and tested in free form. The concentrations used in the assays are not related to cytotoxicity, as cell viability tests defined the non-toxic concentrations.

After preparation, the CME was analyzed by the HPLC-UV chromatography system to confirm the presence of the compounds of interest, according to the method developed by Kerkhoff et al. [13]. A Varian Prostar 325 binary chromatographic system coupled to a Varian Prostar 325 dual UV detector was used, manufactured by Varian Australia Pty Ltd., Mulgrave-Australia, was used. HPLC analysis was performed using a Phenomenex Gemini^®^ C18 reversed-phase analytical column, 5 µm, 110 Å, with dimensions of 250 mm × 4.6 mm. The sample was eluted in a mobile phase composed of ultrapure water (eluent A) and HPLC-grade acetonitrile (eluent B). Elution occurred via a solvent gradient with the following composition variation: 0% B in 0 min to 70% B in 60 min, using a flow rate of 0.80 mL/min. For injection into the chromatographic system, a volume of 20 µL was used. The analysis was monitored by UV, with detection at a wavelength of 296 nm, and the execution time was 60 min.

### 2.2. Subjects

The study comprised 63 healthy individuals, regardless of sex, aged 35 to 50 years, who met the criteria reported by Paoliello-Paschoalato et al. [23]. Blood collection was performed once at the Laboratory of the Research and Teaching Support Center in Health (NUPADS) of the Federal University of Mato Grosso (UFMT) in Sinop and at the Laboratory of Cytology of Biological Fluids and Immunology of the School of Pharmaceutical Sciences of Ribeirão Preto of the University of São Paulo (FCFRP-USP). All participants signed a free and informed consent form to participate in this study, which was approved by the CEP/UFMT-Sinop n. CAAE 5.822.240/2022.

### 2.3. Neutrophil Isolation

Blood (20 mL) was collected from volunteers by venipuncture and immediately diluted in Alsever’s solution (*v*/*v*), which served as an anticoagulant. Neutrophils were isolated by the gelatin method [23,24]. Cell pellets were suspended in Hanks’ balanced salt solution (HBSS) supplemented with 0.1% (*w*/*v*) bovine gelatin (HBSS-gel). Cell preparations contained 80–90% neutrophils with viability greater than 95%, as established by exclusion with Trypan Blue [25].

### 2.4. Preparation of Stimuli

A suspension of ZI (zymosan, 3 mg/mL) in physiological saline solution (0.15 M NaCl) was kept in a boiling water bath (100 °C) for 30 min, then centrifuged at 1500 rpm for 10 min. For each mg of ZI, 0.1 mL of Normal Human Serum (NSH) diluted 1:2 in CFD (Complement Fixation Diluent Buffer) or Heat-Inactivated Human Serum (HIHS) at 56 °C for 30 min was added. SI was used as a negative control for serum complement activity. After incubation in a water bath at 37 °C for 30 min, opsonized zymosan (ZI/NHS) and (ZI/HIHS) were obtained. The suspension was centrifuged under the same conditions as above, and the sediment formed was washed with physiological saline solution under the same conditions [26]. The concentration of the ZI/NHS or ZI/HIHS suspension was adjusted according to the required assay.

### 2.5. Cytotoxicity

With the aim of investigating a possible therapeutic application of CME, its cytotoxicity on human neutrophils was initially evaluated at concentrations of 0.5, 5, and 50 µg/mL based on studies described by Banfi et al. [10]. The cells were incubated for 1 h under culture conditions (37 °C, 5% CO_2_), and cell viability was determined indirectly by quantifying the release of the enzyme lactate dehydrogenase (LDH), as described by Kabeya et al. [27].

### 2.6. Oxidative Metabolism

The oxidative metabolism of neutrophils was evaluated using the luminol-enhanced chemiluminescence (CL) assay according to a methodology adapted from Lucisano-Valim et al. [24]. Briefly, assays were performed with neutrophils (1 × 10^6^ cells/mL) treated for 3 min at 37 °C with CME (0.5 µg, 5 µg, and 50 µg/mL) or its vehicle (0.1% *v*/*v* DMSO) in the presence of luminol (0.1 mM) in 96-well microplates. The cells were stimulated with PMA (10^−7^ μmol/L). The CL was immediately measured in the LB 960 Centro microplate luminometer (Berthold Technologies, Bad Wildbad, Germany) for 15 min at 37 °C. Unstimulated and untreated neutrophils were incubated under the same time and temperature conditions described above.

A second assay of neutrophil oxidative metabolism was performed, specifically to measure the production of the superoxide anion (O_2_·^−^) formed by the reduction of O_2_ by NADPH oxidase, using spectrophotometry by the NBT (nitroblue tetrazolium) method. In this analysis, samples containing neutrophils (4 × 10^6^ cells/mL) were treated with CME (5 µg/mL) or its vehicle (0.1% *v*/*v* DMSO), in the presence of NBT (4 mg/mL), in microtubes. The cells were stimulated with ZI, ZI/NHS, or ZI/HIHS (3 mg/mL) and incubated for 30 min at 37 °C. Next, DMSO was added, the absorbance of the blue formazan product was recorded at 565 nm, and the formazan concentration in μM was determined. O_2_·^−^ was evaluated using unstimulated and untreated neutrophils, incubated under the same time and temperature conditions described above [28].

Finally, the evaluation of neutrophil oxidative metabolism was quantified by means of the production of hypochlorous acid (HOCl), which is formed by the reaction of myeloperoxidase with its substrate, hydrogen peroxide (H_2_O_2_). In this analysis, neutrophils (20 × 10^6^ cells/mL) were incubated with Hanks’ solution and supplemented with taurine (5 mM) and then treated with CME (5 μg/mL) or its vehicle (0.1% *v*/*v* DMSO) in microtubes. Cells were stimulated with ZI/NHS or ZI/HIHS (3 mg/mL) and maintained for 30 min at 37 °C. This was followed by the addition of catalase (2 mg/mL) and centrifugation (2500 rpm, 10 min, 4 °C). The supernatant was transferred to a 96-well plate and incubated with the developing solution containing 20 mM TMB (3,3′,5,5′-tetramethylbenzidine), and after 15 min, the oxidation of TMB was recorded at 630 nm. HOCl was evaluated using unstimulated and untreated neutrophils, incubated under the same time and temperature conditions described above [29].

### 2.7. Degranulation

Neutrophil degranulation induced by f-MLP (N-formyl-methionine-leucyl-phenylalanine) was evaluated using elastase as a marker, according to the methodology modified by Kanashiro et al. [30]. Briefly, neutrophils (5 × 10^6^ cells/mL) were treated with CME (5 µg/mL) or its respective vehicle for 30 min at 37 °C. The cells were stimulated with fMLP (10 mmol/L) and incubated for another 30 min at 37 °C. After centrifugation (2500 rpm, 10 min, 4 °C), the supernatant was transferred to another plate and incubated with the elastase substrate (N-Succinyl-Ala-Ala-Val-Ala p-nitroanilide; 330 μM) for 1 h at 37 °C. The absorbance of the yellow p-nitroaniline product was recorded at 405 nm using a microplate reader.

### 2.8. Production and Release of Neutrophil Extracellular Traps (NETs)

Neutrophils (1 × 10^6^ cells/mL) were treated with CME (5 µg/mL), DPI (diphenyleneiodonium chloride) (1 μM; positive control), or DMSO (0.1% *v*/*v*, vehicle) for 15 min at 37 °C. Cells were stimulated with PMA (10^−7^ μmol/L) and incubated for 4 h (37 °C, 5% CO_2_) under gentle agitation. NET production and release were assessed using quantitative assay protocols (QuantiT™ PicoGreen^®^ dsDNA assay kit, Invitrogen, Thermo Fisher Scientific, Waltham, MA, USA) [31].

### 2.9. Oxidative Stress

For the evaluation of oxidative stress, neutrophils (1 × 10^6^ cells/mL) were incubated with CME 5 μg/mL or controls (HBSS, TRITON X-100, DMSO 0.1%) and maintained in culture at 37 °C, 5% CO_2_ for 4 h [32]. Afterward, the material was centrifuged, and the activities of the antioxidant enzymes, superoxide dismutase (SOD), catalase (CAT), glutathione-S-transferase (GST), the non-oxidant enzyme reduced glutathione (GSH), and the generation of protein carbonylation were evaluated in the supernatant. SOD activity was measured using the adapted method of Misra and Fridovich [33], which consisted of spectrophotometric monitoring of the adrenochrome formation rate at 480 nm. The results of the analysis were expressed in international units of IU SOD/mg protein. CAT activity was analyzed according to Nelson and Kiesow [34]. The variation in hydrogen peroxide absorbance in this method allowed the calculation of CAT activity in µmol H_2_O_2_/min/mg protein. GST activity was determined using CDNB (1-chloro-2,4-dinitrobenzene) [35] to determine enzymatic activity, expressed in μmol GS-DNB/min/mg protein. The non-enzymatic antioxidant reduced glutathione (GSH) was also analyzed using the method adapted from Sedlack and Lindsay [36]. The analysis involved comparing the analytical signal of the samples with a calibration curve prepared with solutions of known concentrations (GSH). GSH levels were expressed in μmol GSH/mg protein. The concentration of carbonylated proteins (PCO) was determined by the method described by Colombo et al. [37] and expressed in nmol PCO/mg protein. The protein content of the supernatant was also determined for the calculation of the other biochemical parameters. The Bradford method [38] was used, which employs the Coomassie Brilliant Blue dye, which interacts with the proteins present in the sample. The results were obtained by comparison with a standard curve of bovine serum albumin.

### 2.10. Statistical Analysis

Initially, the data were evaluated using the Kolmogorov–Smirnov normality test. For data that did not meet the normality test, the Kruskal–Wallis (K.W.) test combined with Dunn’s post hoc test was performed. Otherwise, a one-way ANOVA test followed by Tukey’s post hoc test was performed. The minimum acceptable significance level was *p* < 0.05. Results are expressed as median and interquartile range (for K.W.) or mean and standard deviation (ANOVA). Graphs were log-transformed, and the results were analyzed using the GraphPad Prism 8.0^®^ statistical program (GraphPad Software Inc., San Diego, CA, USA).

## 3. Results

### 3.1. CME Analysis by HPLC-UV

**Figure 1:**
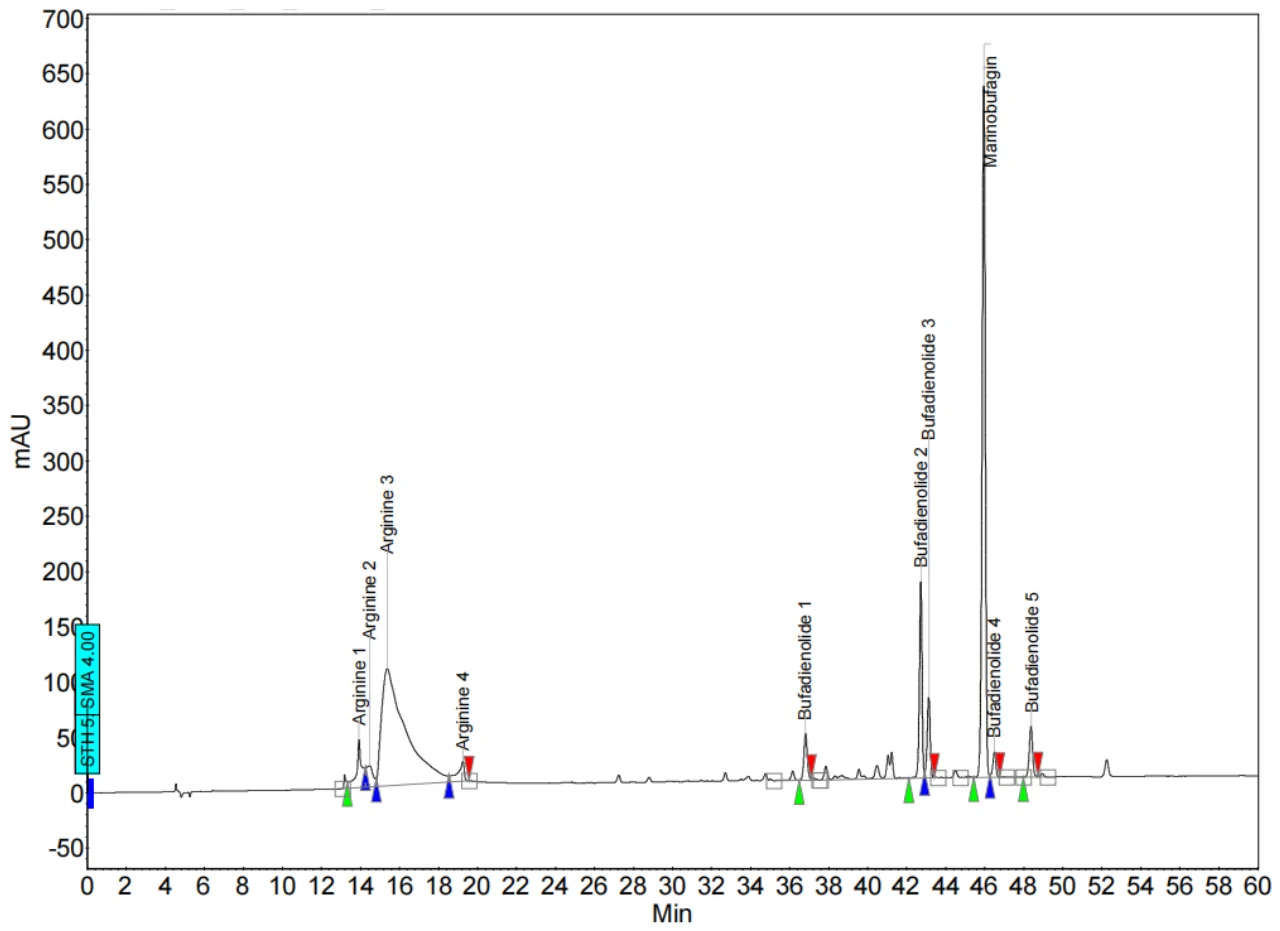
Chromatogram obtained from the compounds.

As can be seen in the chromatogram in Figure 1, the chromatographic band called “Arginine” corresponds to arginine and represents 45.54% of the sample. The percentage of each compound in the sample is calculated through the integration of the peaks performed by the data system software. This identification was carried out by Pelissari et al. (2021) [3] using High-Performance Liquid Chromatography coupled to a detector by ultraviolet spectrophotometry techniques (HPLC-UV). It can be observed that the peaks with retention times of 42.72 min and 48.37 min, named “Bufadienolide 1” and “Bufadienolide 3”, respectively, correspond to two bufadienolides and represent 11.04% of the total sample. It was not possible to confirm the chemical identity of the bufadienolides analyzed due to the lack of standard reference samples. From analyses of the peak with a retention time of 45.95 min, called “Marinobufagin”, it is possible to state that the identified compound corresponds to the bufadienolide marinobufagin and represents 35.48% of the sample composition. This identification was made by comparison with a previously existing marinobufagin standard.

### 3.2. Cytotoxicity Determination

**Figure 2:**
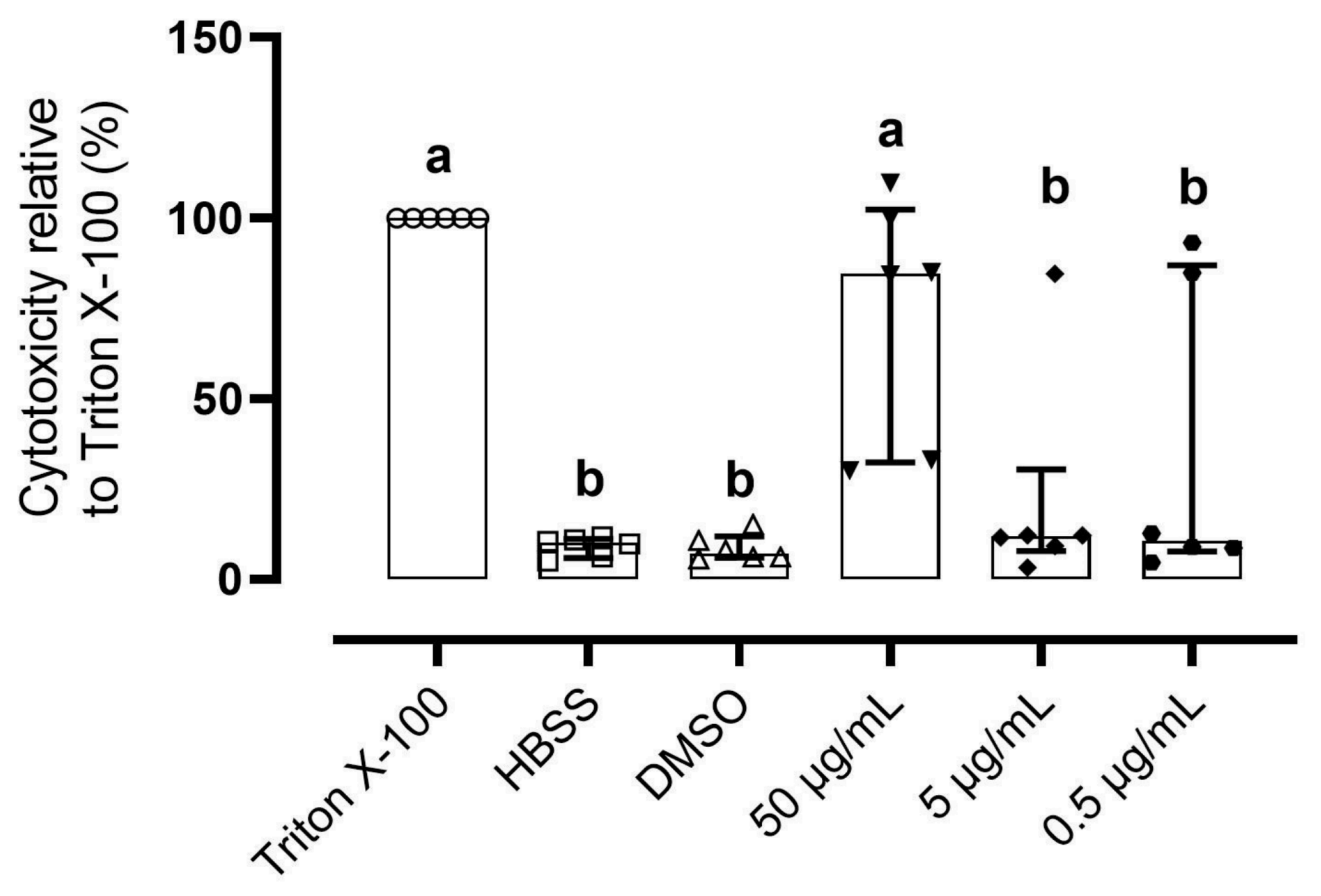
Cytotoxicity of CME (50, 5, and 0.5 µg/mL) on peripheral blood neutrophils from healthy individuals, determined by lactate dehydrogenase activity and expressed relative to Triton X-100-induced cell lysis, defined as 100%. Results are expressed as the median and interquartile range (n = 6 in duplicate). Kruskal–Wallis test was used, followed by Dunn’s multiple-comparison test, with each treatment compared with the control (100% lysis); *p* < 0.05. Data with different letters indicate a statistically significant difference. DMSO, dimethyl sulfoxide; HBSS, Hanks’ balanced salt solution.

CME affected neutrophil viability, as assessed by the LDH release assay. Cytotoxicity was calculated relative to Triton X-100-induced cell lysis, which was considered 100%. No significant differences in cell viability were observed between the groups treated with the extract at 0.5 and 5 µg/mL or with the solvent and the negative control (HBSS/Hanks). However, treatment with the extract at 50 µg/mL resulted in high cytotoxicity, similar to that induced by the positive control (Triton X-100), suggesting a potential cytotoxic effect of the CME at this concentration (Figure 2).

Thus, under the experimental conditions evaluated, CME presented concentrations considered both toxic and non-toxic to peripheral blood neutrophils from healthy individuals, maintained under incubation at 37 °C for 1 h, were used. Based on these results, a subsequent functional experiment (oxidative metabolism) was conducted using concentrations of 0.5 and 5 µg/mL, due to the absence of cytotoxicity.

### 3.3. CME Modulates Neutrophil Oxidative Metabolism

**Figure 3:**
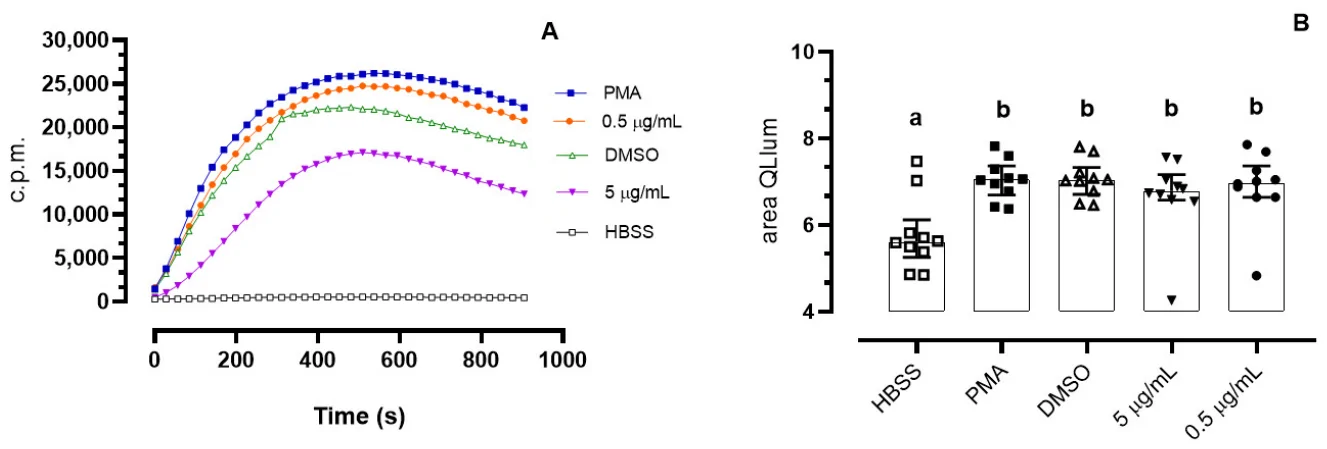
ROS production by peripheral blood neutrophils from healthy individuals treated with CME (5 and 0.5 µg/mL) and stimulated by PMA, measured by luminol-dependent chemiluminescence of neutrophils: (**A**) Representative kinetic profile of neutrophils from an individual. (**B**) Area under the curve of the chemiluminescence profile (n = 10 in duplicate). Results are expressed as the median and interquartile range. Kruskal–Wallis test was used, followed by Dunn’s multiple-comparisons test, with each treatment compared with the positive control (PMA); *p* < 0.05. Data with different letters show a statistically significant difference. CPM, photon counts per minute; DMSO, dimethyl sulfoxide; HBSS, Hanks’ balanced salt solution; PMA, myristoylphorbol acetate.

First, assays were performed to evaluate the modulating effect of CME (0.5 and 5 μg/mL) on ROS in PMA-stimulated neutrophils. Compared to the control, the CME concentrations tested did not show a significant difference (Figure 3A,B).

**Figure 4:**
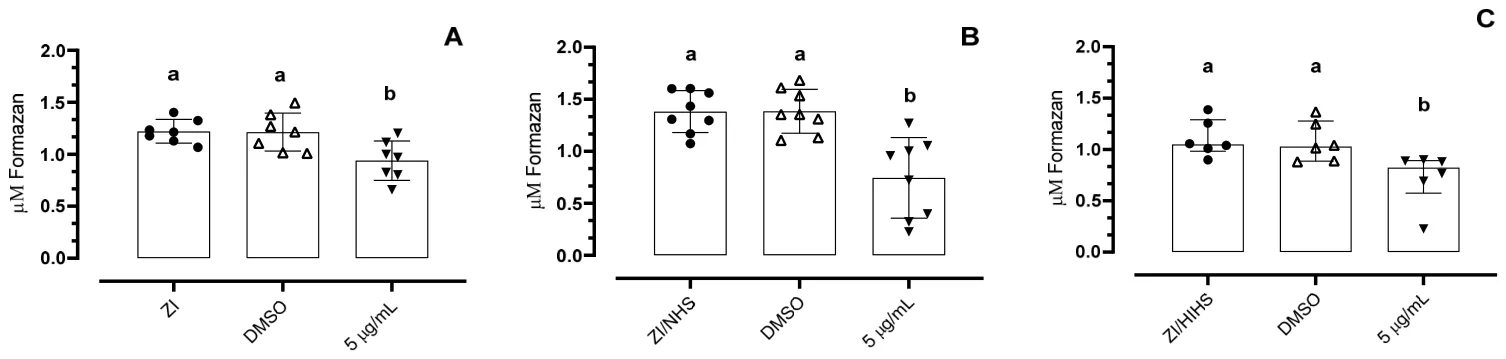
Quantification of superoxide anion (O_2_·^−^) in peripheral blood neutrophils from healthy individuals previously incubated with CME (5 µg/mL) and stimulated: (**A**) ZI (n = 7 in duplicate). (**B**) ZI/NHS (n = 8 in duplicate). (**C**) ZI/HIHS (n = 6 in duplicate). Results are expressed as means and standard deviations. ANOVA test followed by Tukey’s post hoc test; *p* < 0.05. Data with different letters show a statistically significant difference. DMSO, dimethyl sulfoxide.

It was observed that both concentrations (0.5 and 5 μg/mL) did not lead to modulation of function. Subsequently, the NBT reduction assay was performed to evaluate the inhibitory effect of CME only at a concentration of 5 μg/mL on the generation of O_2_·^−^ by neutrophils stimulated with ZI, ZI/NHS, or ZI/HIHS (Figure 4A–C, respectively). Compared to the control group, treatment with CME demonstrated a significant inhibitory effect on O_2_·^−^ production when neutrophils were stimulated in both tests.

**Figure 5:**
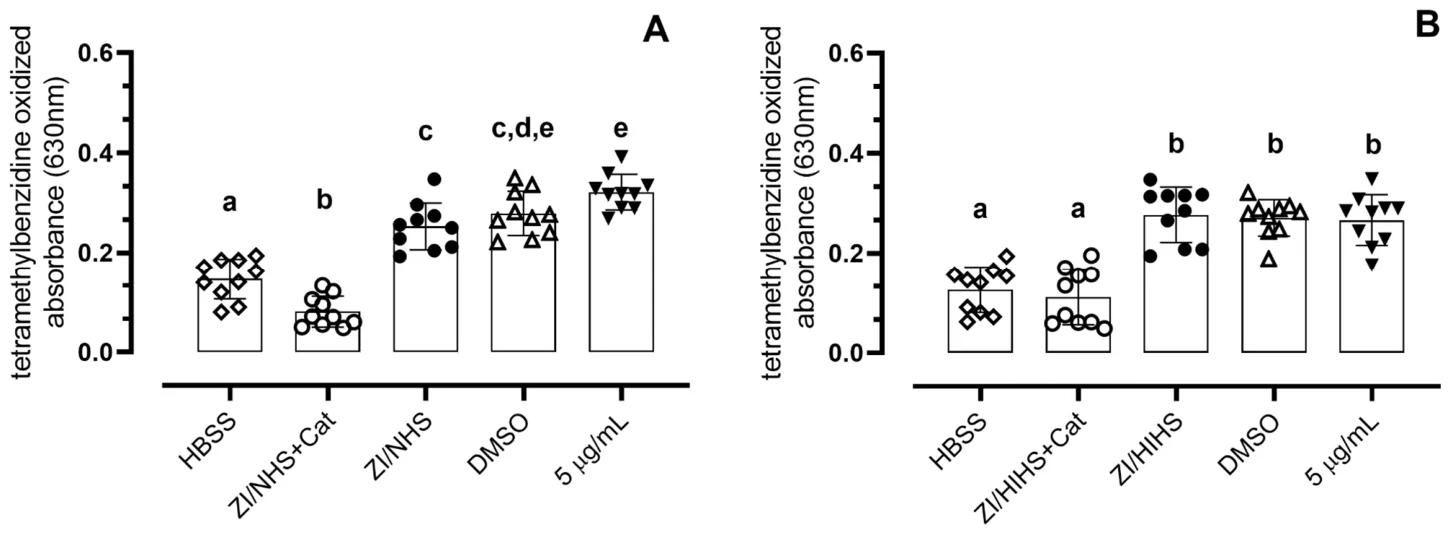
Oxidation of TMB and percentage of TMB oxidized by taurine–chloramine from HOCl production by MPO of peripheral blood neutrophils from healthy individuals treated with CME (5 µg/mL): (**A**) ZI/NHS (n = 10 in duplicate). (**B**) ZI/HIHS (n = 10 in duplicate). Results are expressed as means ± standard deviation. ANOVA followed by Tukey’s post hoc test; *p* < 0.05. Data with different letters show a statistically significant difference. Cat, catalase; DMSO, dimethyl sulfoxide; TMB, 3,3′,5,5′-tetramethylbenzidine; HBSS, Hanks’ balanced salt solution.

Furthermore, oxidative metabolism was assessed by myeloperoxidase activity through TMB oxidation, and inhibition of hypochlorous acid production was observed. Neutrophils treated with CME (5 μg/mL) were stimulated by ZI/NHS or ZI/HIHS, verifying the ability to inhibit hypochlorous acid production. When compared to the ZI/NHS control, the extract did not show an inhibitory effect (Figure 5A,B).

### 3.4. CME Modulates Neutrophil Degranulation

**Figure 6:**
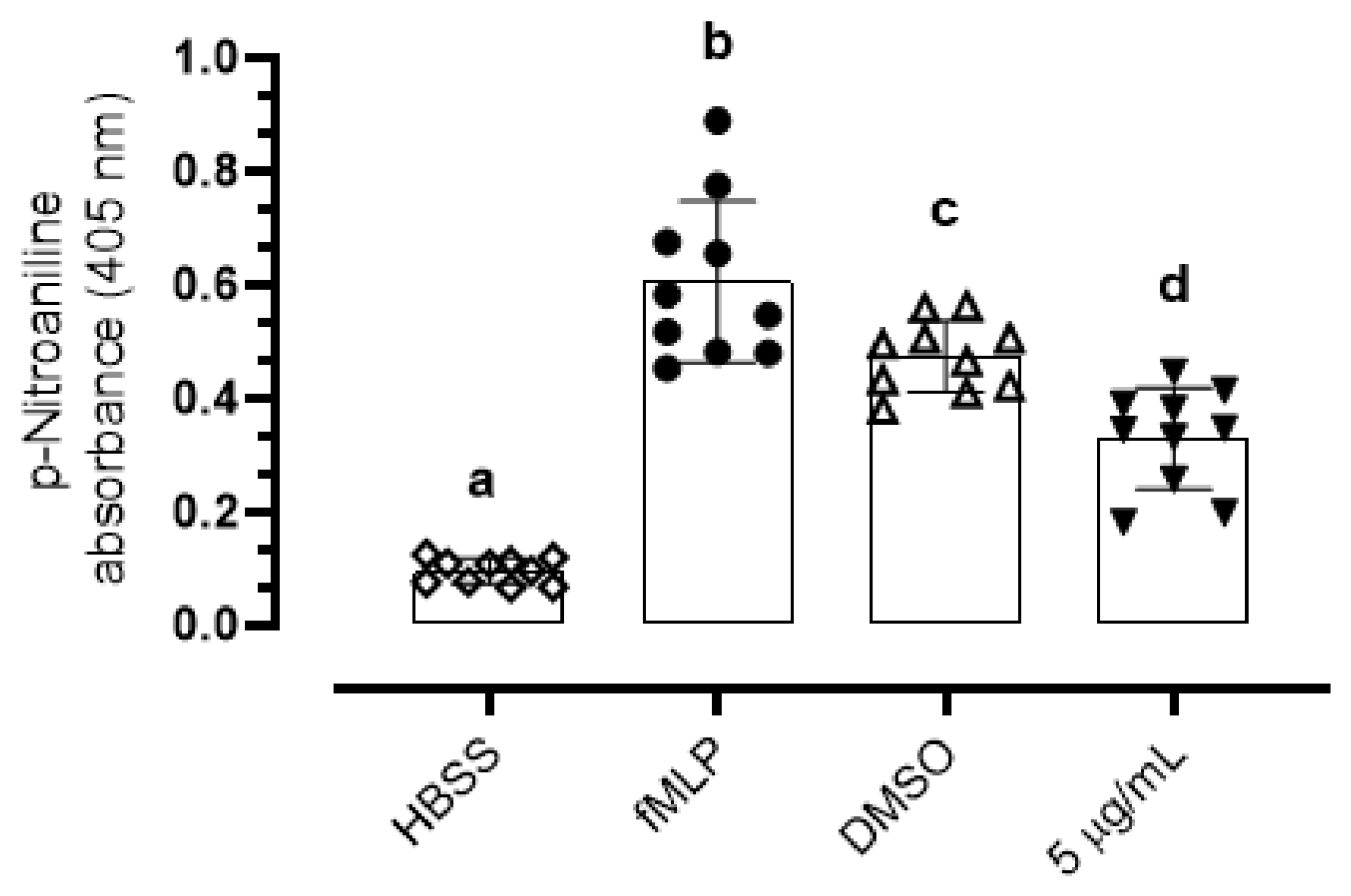
Evaluation of elastase enzyme activity released from peripheral blood neutrophils of healthy individuals previously incubated with CME (5 µg/mL) and stimulated by fMLP. Results are expressed as means ± standard deviation. ANOVA followed by Tukey’s post hoc test; *p* < 0.05 (n = 10 in duplicate). Data with different letters show a statistically significant difference. DMSO: dimethyl sulfoxide; fMLP: N-formyl-methionyl-leucyl-phenylalanine; HBSS: Hanks’ balanced salt solution.

Since the CME modulates O_2_·^−^ production, as assessed by the NBT assay, its effect on neutrophil degranulation was evaluated by measuring the activity of elastase released into the supernatant. Elastase enzyme activity was assessed through the degradation of the substrate N-SUCCINYL-ALA-ALA-VAL-ALA based on p-nitroaniline production. In the presence of the extract, significantly lower elastase activity was observed in the neutrophil supernatant compared with the positive control (fMLP) (Figure 6).

### 3.5. CME Does Not Interfere with NET Formation

**Figure 7:**
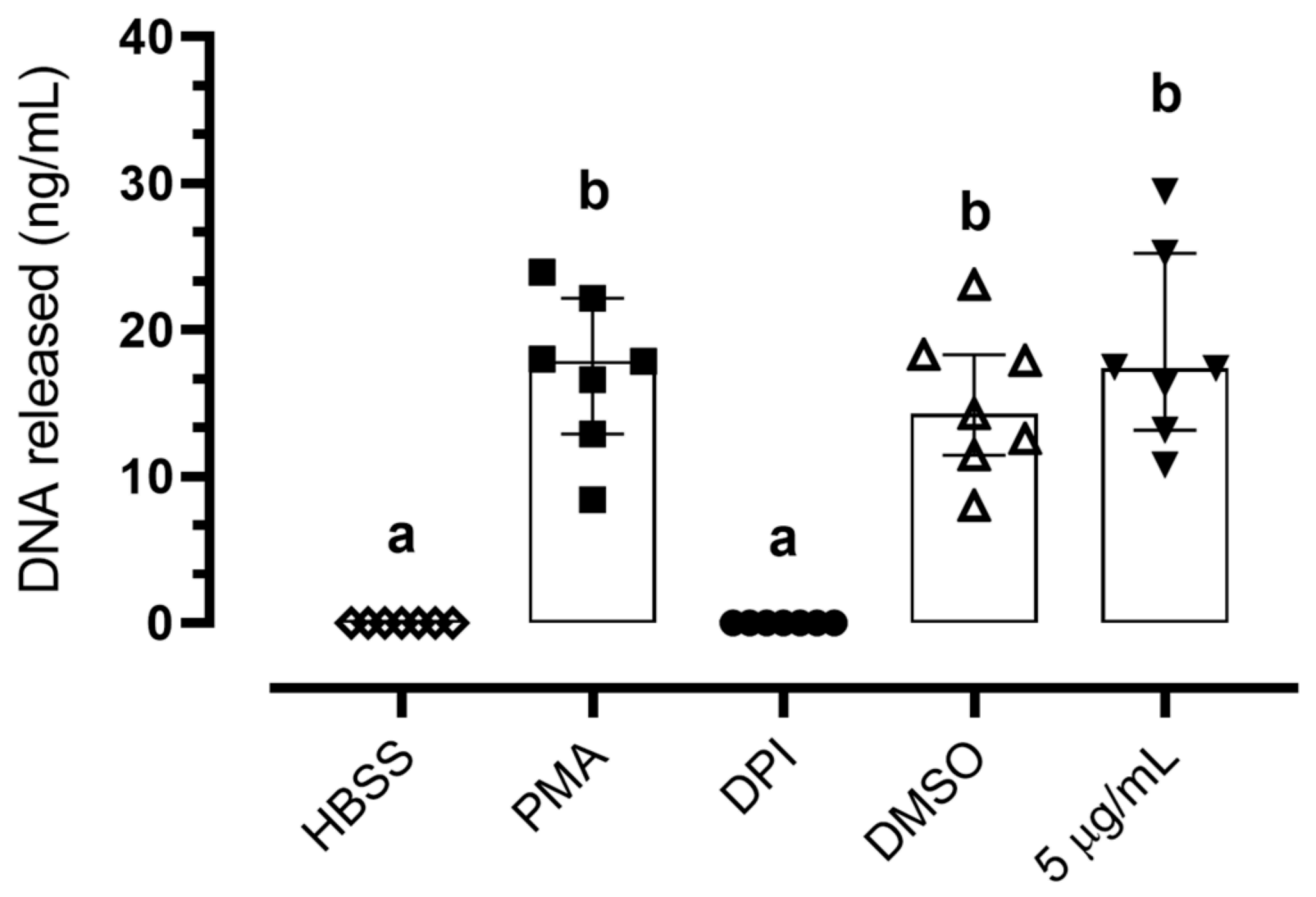
Quantification of DNA release from peripheral blood neutrophils of healthy individuals incubated with CME (5 µg/mL), stimulated by PMA (n = 7 in duplicate). Data with different letters show a statistically significant difference. Results are expressed as the medians and interquartile ranges. Kruskal–Wallis test followed by Dunn’s post hoc test; *p* < 0.05. DMSO, dimethyl sulfoxide; DNA, deoxyribonucleic acid; DPI, diphenylene iodonium; HBSS, Hanks’ balanced salt solution; PMA, phorbol myristate acetate.

Regarding NET release, CME (5 μg/mL) was not able to inhibit it in neutrophils from healthy individuals stimulated with PMA (Figure 7), showing a result similar to the positive control (PMA).

### 3.6. CME Partially Modulates Oxidative Stress

The results did not demonstrate statistically significant differences among the groups regarding SOD activity. Regarding CAT activity, an increase was observed in the group treated with Triton X-100; however, no statistical differences were found in the other experimental groups. For GST activity, a significant increase was observed in the vehicle control groups (0.1% DMSO) and those treated with CME when compared to the negative and positive controls. Regarding GSH, treatment with CME promoted an increase in its release in the supernatant, compared to the controls. Finally, the levels of PCO did not show statistically significant differences between the evaluated groups (Table 1).

## 4. Discussion

Through HPLC analysis, it was possible to confirm the presence of several bufadienolides in the CME, with 3 of them being the predominant components of the CME, accounting for more than 50%. It should be noted that, in previous studies, we identified that all compounds appearing in the chromatogram with retention times between 26 and 55 min correspond to steroidal compounds. This is due to the fact that these compounds are the least polar of the venom constituents, and since the analyses were performed using a reversed-phase system, these are the last to be eluted from the column [3,12,13].

The cytotoxicity of natural products and animal toxins is often dependent on the concentration used, which is a fundamental aspect for interpreting their biological effects. Santangelo et al. [39] evaluated the cytotoxic potential of the CME of *R. marina* in peripheral human monocytes and demonstrated that toxicity occurred in a concentration-dependent manner. These authors observed that high concentrations of the extract (1000, 500, 250, 125, and 31.25 µL/mL) significantly reduced cell viability, exhibiting behavior similar to the positive cell lysis control (Triton X-100). In contrast, concentrations of 62.5, 15.63, and 7.8 µL/mL did not significantly reduce monocyte viability and were considered non-cytotoxic. In this context, the use of low concentrations of the extract, such as that employed in the present study (5 µg/mL), proves to be adequate for investigating possible immunomodulatory effects in human neutrophils, minimizing interferences resulting from direct cytotoxicity, since these are cells that live together in the blood. These results reinforce the importance of prior determination of cell viability for defining experimentally safe concentrations, allowing changes observed in functional parameters to be attributed predominantly to cell modulation and not to cell death induced by the treatment.

In this study, we investigated the ability of CME to modulate neutrophil effector functions in healthy individuals using assays that evaluate different stages of ROS generation. First, neutrophils were treated with CME and then stimulated with PMA, a potent intracellular activator of protein kinase C (PKC). ROS production was assessed using a luminol-dependent chemiluminescence assay. Luminol is a luminescent probe that enables sensitive detection of ROS generated both intracellularly and extracellularly, including oxidants associated with the myeloperoxidase (MPO) system. No significant difference in ROS production was observed in neutrophils following CME treatment. PMA is an artificial stimulus widely used to investigate neutrophil activation because it directly activates protein kinase C (PKC), mimicking the action of the endogenous second messenger diacylglycerol. This activation induces several neutrophil effector functions, including ROS production and degranulation [40]. Therefore, the absence of a significant effect of CME on ROS production following PMA stimulation suggests that, under the conditions evaluated, CME did not significantly alter the oxidative response elicited by direct PKC activation. However, this finding does not exclude effects on other intracellular mechanisms or on signaling pathways activated by receptor-mediated stimuli.

Second, superoxide anion generation was specifically evaluated using the NBT assay. Neutrophils were stimulated in the presence of NBT, a yellow tetrazolium salt that is reduced by superoxide anion to form an insoluble blue formazan product within the cells, which can be quantified by spectrophotometry. In this assay, neutrophils were stimulated with zymosan, either untreated or serum-treated, under conditions involving different modes of particle opsonization as receptor-mediated stimuli. CME significantly reduced superoxide anion generation under these conditions. Zymosan is widely used as an experimental stimulus to investigate neutrophil activation and oxidative responses. Its complex cell wall contains carbohydrate structures that can be recognized by multiple pattern recognition receptors (PRRs) on phagocytes. In addition, when opsonized with serum components, zymosan can engage opsonic receptors, including Fcγ receptors (FcγR) and complement receptors (CRs), thereby activating distinct and potentially convergent signaling pathways involved in neutrophil activation. Consequently, zymosan stimulation can induce a robust oxidative response involving multiple cellular mechanisms. In the present study, zymosan was therefore used as a physiologically relevant particulate stimulus to evaluate the ability of CME to modulate neutrophil ROS production. This approach is consistent with previous studies showing that neutrophils produce ROS following stimulation with zymosan, either through recognition by pattern recognition receptors or, following opsonization, through interactions with FcγRs and complement receptors, ultimately leading to NADPH oxidase activation and reactive oxygen species generation [41]. Although the experimental conditions do not allow the modulation of pathways mediated by specific receptors to be directly attributed, zymosan itself, a polysaccharide, provides ligands for Toll-like receptors. In addition, serum-treated zymosan provides ligands for CR expressed on neutrophils and may be recognized by natural immunoglobulins that bind to carbohydrates present in zymosan, thereby potentially engaging FcR on neutrophils. Thus, the possibility that the CME may modulate signaling pathways involving these receptors could be considered, providing opportunities for future investigations into the mechanisms underlying these effects.

In addition, the absence of a significant effect under PMA stimulation does not, by itself, demonstrate that the CME does not interfere with intracellular mechanisms downstream of membrane receptors. Importantly, because individual receptor pathways were not selectively blocked or directly investigated, the present findings do not allow a specific receptor to be identified as the molecular target of CME. Therefore, these findings may be interpreted as suggesting a modulatory effect on stimulus-induced ROS generation, without necessarily implicating a specific receptor or signaling pathway as a molecular target of the CME.

Furthermore, the effect of CME on neutrophil oxidative metabolism was also evaluated by measuring hypochlorous acid (HOCl) production. Superoxide anion is the first ROS generated during neutrophil oxidative metabolism, produced by the activity of the NADPH oxidase complex in response to phagocytic stimuli. It is rapidly converted into hydrogen peroxide, which is subsequently used by myeloperoxidase (MPO) to oxidize chloride ions, resulting in the formation of HOCl [42]. HOCl is an important microbicidal ROS involved in the antimicrobial activity of neutrophils. CME pretreatment had no significant effect on HOCl production by neutrophils subsequently stimulated with zymosan, either untreated or serum-treated. Thus, measuring HOCl production provides an additional perspective on the complex process underlying neutrophil oxidative metabolism. The lack of a significant effect of CME on HOCl production suggests that, under the experimental conditions evaluated, the extract did not significantly alter this downstream oxidative response, despite its effects on other parameters of neutrophil oxidative metabolism.

Considering that the effector mechanisms used by neutrophils to eliminate infectious agents depend, directly or indirectly, on intracellular redox balance, changes in ROS production may influence other important cellular functions, including phagocytosis, degranulation, NET formation, chemotaxis, and intracellular microbicidal activity. Furthermore, the intracellular redox state plays an important role in regulating signaling pathways involved in neutrophil activation, survival, and apoptosis, potentially influencing both the intensity and resolution of the inflammatory response [43,44,45,46]. In this context, partial modulation of these responses may be of particular interest in inflammatory conditions characterized by exacerbated neutrophil activation, in which excessive production of oxidative mediators may contribute to tissue damage [19].

When we analyzed the effect of CME on degranulation, significantly lower elastase activity was observed in the neutrophil supernatant previously treated with the extract compared with the positive control (fMLP). fMLP acts on neutrophils via a specific receptor called the formylated peptide receptor, which belongs to the G-protein-coupled receptor family. The fMLP stimulation of neutrophils activates a wide variety of intracellular signaling pathways mediated by phosphatidylinositol-specific phospholipase C, phospholipase A2, phospholipase D, phosphatidylinositol 3-kinase, and mitogen-activated protein kinases to induce various cellular functions [47]. Among the main responses elicited by FMLP in neutrophils are the generation of reactive oxygen species and the release of lysosomal enzymes [48]. Degranulation is functionally related to oxidative metabolism, since the mobilization and release of granular enzymes depend, at least in part, on the ionic influx associated with the activation of the NADPH oxidase complex and the production of the superoxide anion [15].

Previous studies have shown that antioxidant compounds capable of interfering with NADPH oxidase and MPO activity can modulate elastase release and NET formation, contributing to a reduction in the intensity of the inflammatory response [15,49,50,51]. Our results show that treatment of neutrophils with CME resulted in a significant difference in superoxide generation, as indirectly assessed by the NBT assay, while no significant differences were observed in HOCl production or NET formation. A significant difference was also observed in elastase activity measured in the neutrophil supernatant. These findings suggest that CME may modulate neutrophil oxidative and degranulation responses under the experimental conditions evaluated. However, the present assays do not allow us to determine whether the observed differences resulted from quantitative changes in the production or release of the measured components or from qualitative changes in their activity. Because NADPH oxidase activity was not directly measured, the observed difference in superoxide generation cannot be specifically attributed to the inhibition or activation of the NADPH oxidase complex. Similarly, the difference in elastase activity cannot be directly interpreted as altered enzyme release, as the assay did not distinguish between changes in enzyme release and changes in enzymatic activity. Further mechanistic studies, including cell-free assays, will be necessary to determine the processes underlying the effects observed with CME. These findings suggest that the partial modulation of oxidative metabolism observed following CME treatment may be associated with changes in neutrophil degranulation, as reflected by the difference in elastase activity measured in the supernatant. Thus, although differences were observed in specific parameters, the mechanisms underlying these differences remain to be determined.

The relevance of these findings becomes particularly interesting in the context of chronic inflammatory diseases, such as rheumatoid arthritis, in which neutrophils exhibit increased susceptibility to activation and release enzymes capable of promoting tissue degradation, including elastase [19,52]. In addition to increased oxidative responses and greater expression of Toll-like and complement receptors in peripheral blood neutrophils, patients with active rheumatoid arthritis, even when undergoing methotrexate monotherapy, may present increased serum levels of chemotactic factors and exacerbated leukocyte recruitment to the inflamed synovium [43,53]. Persistent infiltration and activation of neutrophils contribute significantly to the tissue damage observed in autoimmune diseases, as these cells release ROS, proteases, and NETs capable of perpetuating the inflammatory process. In this context, the ability of CME to modulate selected neutrophil responses observed in the present study may be of interest for further investigation as a potential approach to modulate neutrophil-mediated inflammatory processes. However, since the experiments in the present study were performed using neutrophils from healthy individuals under in vitro conditions, further investigations using patient-derived cells and inflammatory models are needed to determine the pathophysiological relevance of these findings and to further explore the immunomodulatory potential of CME.

Bala et al. [54] associated the pathophysiology of rheumatoid arthritis with oxidative stress and highlighted the potential of bioactive molecules derived from natural products to modulate the activity of different inflammatory cells. Similarly, corticosteroid compounds have well-established anti-inflammatory activity in vivo, associated with reduced leukocyte infiltration, production of inflammatory mediators, and oxidative stress [23]. Conventional glucocorticoids also exert immunomodulatory effects on neutrophils, including the suppression of chemotaxis, cell adhesion, degranulation, and ROS release, in addition to influencing mechanisms related to cell survival [51].

Although CME modulated selected neutrophil responses in vitro in neutrophils from healthy individuals, the findings of the present study do not yet allow us to determine the pathophysiological context, disease stage, or therapeutic setting in which its potential as a natural anti-inflammatory compound could be further investigated in autoimmune diseases. Therefore, further studies using neutrophils from patients and experimental models of inflammation are needed to clarify the potential anti-inflammatory relevance of CME and the mechanisms underlying its immunomodulatory effects.

During physiological imbalance, such as that observed in autoimmune diseases, the antioxidant system is constantly challenged by the exacerbated production of reactive species associated with chronic inflammation. This system comprises enzymatic antioxidants, such as SOD, CAT, GST, and GPx, as well as endogenous and exogenous non-enzymatic antioxidants, including GSH, antioxidant vitamins, and other bioactive compounds, which act in an integrated manner to maintain cellular redox homeostasis [20,53]. In this context, considering the central role of neutrophils in the inflammatory response and their ability to produce high levels of ROS during oxidative metabolism, the present study evaluated the activity of important antioxidant enzymes involved in maintaining redox homeostasis following in vitro treatment with CME. Among these, SOD activity was analyzed, as SOD is considered a first line of antioxidant defense against ROS and is an essential component of the endogenous antioxidant system, catalyzing the conversion of superoxide anion into H_2_O_2_. CAT activity was also evaluated, as this enzyme catalyzes the degradation of H_2_O_2_ into water and molecular oxygen, thereby preventing the accumulation of potentially cytotoxic oxidizing metabolites [20]. In neutrophil cultures treated with CME, no statistically significant differences in SOD or CAT activity were observed compared with the control groups. This finding may suggest that, under the experimental conditions evaluated, CME did not significantly affect these enzymatic antioxidant parameters involved in the primary detoxification of ROS in neutrophils, although a possible effect on other redox pathways or cellular effector functions associated with neutrophil activation, such as degranulation, cannot be ruled out [41,55,56]. Furthermore, a significant difference in GST activity was observed. GST comprises a group of enzymes widely recognized for their role in the detoxification of xenobiotics and in cellular protection against oxidative stress. These enzymes catalyze the conjugation of GSH with potentially toxic electrophilic compounds, favoring their neutralization, increasing their solubility, and facilitating their subsequent elimination from the organism, in addition to contributing to protection against oxidative damage induced by ROS [57]. In the present study, GST activity differed significantly in the groups treated with CME compared with the positive control, suggesting that GST-related mechanisms may be involved in the cellular response to redox imbalance under the experimental conditions evaluated. These findings partially corroborate those reported by Veselinovic et al. [56], who also observed alterations in antioxidant parameters, suggesting that mechanisms associated with the glutathione system may contribute to cellular adaptation to oxidative stress. Changes in GST activity have also been described in inflammatory and autoimmune diseases, in which alterations in this enzyme have been interpreted as a possible compensatory response to increased production of reactive species and consumption of endogenous antioxidants [58]. Considering the role of GST in the conjugation of reduced glutathione (GSH) with potentially toxic electrophilic compounds, the difference observed in GST activity may reflect an alteration in cellular mechanisms involved in the response to oxidative stress.

GSH acts directly in the neutralization of free radicals and the detoxification of oxidizing metabolites, in addition to serving as a cofactor for antioxidant enzymes, particularly GPx and GST, thereby contributing to cellular protection against ROS-induced damage [59,60]. In the present study, a significant increase in GSH concentrations was observed in the group treated with CME compared with the control groups, suggesting a possible modulatory effect of CME on antioxidant mechanisms associated with cellular redox balance. The concomitant differences observed in GST activity and GSH concentrations may indicate an effect of CME on components of the glutathione-dependent antioxidant system under the experimental conditions evaluated. However, these findings do not establish whether the observed increase in GSH resulted from increased synthesis, reduced consumption, altered recycling, or other mechanisms affecting cellular glutathione homeostasis.

Considering that increased ROS production during inflammatory processes can contribute significantly to tissue damage, protein carbonylation was also evaluated as a widely recognized marker of oxidative damage to cellular proteins. Protein carbonylation results from the irreversible oxidation of amino acid residues and can reflect the extent of redox imbalance and oxidative damage [61,62,63]. However, in the present study, no significant differences were observed in the levels of carbonylated proteins following treatment with CME. Although this finding does not allow us to infer a direct protective effect against protein oxidative damage under the experimental conditions evaluated, the absence of increased protein carbonylation, together with the differences observed in parameters related to oxidative metabolism and the glutathione system, may be compatible with the maintenance of redox homeostasis without detectable protein oxidative damage in the neutrophils studied.

Taken together, the findings of the present study suggest an immunomodulatory potential of the CME of *R. marina*, with selective effects on effector functions of human neutrophils associated with inflammatory responses. Differences were observed in parameters related to oxidative metabolism and elastase activity, whereas no significant differences were observed in HOCl production or NET formation. In parallel, differences in GST activity and increased GSH concentrations were observed, while no significant differences were detected in SOD or CAT activities or in protein carbonylation. These findings suggest that CME may modulate selected components of neutrophil effector functions and the glutathione-dependent antioxidant system under the experimental conditions evaluated. However, the present findings do not establish whether these effects result from changes in specific signaling pathways, enzyme activity, production or release of the measured components, or other cellular mechanisms. Overall, these results support further investigation of the CME of *R. marina* as a potential source of bioactive molecules that could be explored in the context of inflammatory conditions characterized by neutrophil activation and oxidative imbalance. Nevertheless, since the experiments were conducted in vitro using neutrophils from healthy individuals, further studies using patient-derived cells and experimental models of inflammation are needed to determine the pathophysiological relevance of these findings and to clarify the mechanisms underlying the observed effects and the immunomodulatory potential of CME.

## 5. Conclusions

The preliminary results reported here suggest that CME may modulate selected neutrophil functions involved in the early stages of the inflammatory response. The observed effects on parameters related to oxidative metabolism, elastase activity, and the glutathione-dependent antioxidant system support further investigation of CME as a potential source of bioactive molecules that could be explored as an adjunctive approach in inflammatory conditions in which neutrophils play a significant role. In this sense, further studies may explore whether CME could have potential as a therapeutic adjuvant in the management of inflammatory conditions, including whether its use could contribute to reducing the need for conventional treatments, such as disease-modifying antirheumatic drugs and non-steroidal anti-inflammatory drugs (NSAIDs), or to mitigating tissue damage associated with increased neutrophil activation. However, further investigations using patient-derived cells and experimental models of inflammation are needed to determine the pathophysiological relevance of these findings and to clarify the mechanisms underlying the observed effects.

## Figures and Tables

**Table 1:** Evaluation of redox state parameters from peripheral blood neutrophils of healthy individuals previously incubated with CME (5 µg/mL).

Marker	HBSS	TRITON X-100	DMSO 0.1%	CME 5 μg/mL
SOD (IU SOD/mg protein)	1.755; 1.389–2.048 a	1.620; 1.439–1.757 a	1.997; 1.513–2.110 a	1.641; 1.328–2.164 a
CAT (μmol H_2_O_2_/min/mg protein)	1.460; 1.342–1.560 a	1.775; 1.627–1.989 b	1.704; 1.186–1.835 ab	1.734; 1.372–2.108 ab
GST (μmol GS-DNB/min/mg protein)	2.064; 1.989–2.254 ab	1.882; 1.720–2.087 a	2.397; 2.053–2.435 b	2.439; 2.109–2.608 b
PCO (μmol/min/mg protein)	1.460; 1.342–1.560 a	1.24; 1.681–2.068 b	1.704; 1.186–1.835 ab	1.734; 1.372–2.108 ab
GSH (μmol GSH/mg protein)	2.119 ± 0.1684 a	1.957 ± 0.1138 a	2.156 ± 0.1321 a	2.400 ± 0.0527 b

Data expressed as median and interquartile range for SOD, CAT, GST, and PCO (n = 10 in duplicate); Kruskal–Wallis test followed by Dunn’s test. For GSH, data are expressed as mean ± standard deviation; ANOVA followed by Tukey’s test. Data were log-transformed. Data with the same letters in each row are not statistically different (*p* > 0.05). CAT, catalase; CME, crude methanolic extract; DMSO, dimethyl sulfoxide; GSH, reduced glutathione; GST, glutathione-S-transferase; HBSS, Hanks’ balanced salt solution; IU, international unit; PCO, protein carbonyls; SOD, superoxide dismutase.

## Data Availability

The data that support the findings of this study are available from the corresponding author upon reasonable request.

## Abbreviations

The following abbreviations are used in this manuscript:

ABAM-UFMT	Biological Collection of Southern Amazonia at the Federal University of Mato Grosso
CAAE	Certificate of Presentation for Ethical Review
CAT	Catalase
CDNB	1-Chloro-2,4-Dinitrobenzene
CEP/UFMT	Research Ethics Committee of the Federal University of Mato Grosso
CFD	Diluent Buffer for Fixation of Supplement
CL	Chemiluminescence
CME	Crude Methanolic Extract
CR1	Complement Receptor 1
DMSO	Dimethyl Sulfoxide
DPI	Diphenyleneiodonium chloride
FAPEMAT	Mato Grosso Research Support Foundation
FCFRP-USP	School of Pharmaceutical Sciences of Ribeirão Preto, University of the State of São Paulo
fMLP	N-Formyl-Methionine-Leucyl-Phenylalanine
GPx	Glutathione Peroxidase
GSH	Reduced Glutathione
GST	Glutathione S-Transferase
H_2_O_2_	Hydrogen Peroxide
HBSS	Hanks’ Balanced Salt Solution
HOCl	Hypochlorous Acid
IBAMA	Brazilian Institute of Environment and Renewable Natural Resources.
KW	Kruskal–Wallis
LDH	Lactate Dehydrogenase
MPO	Myeloperoxidase
NaCl	Sodium Chloride
NADPH	Nicotinamide Adenine Dinucleotide Phosphate
NBT	Nitroblue Tetrazolium
NETs	Neutrophil Extracellular Traps
NUPADS	Research and Teaching Support Center in Health
O_2_·^−^	Superoxide Anion
PCO	Carbonyl Proteins
PMA	Myristoylphorbol Acetate
ROS	Reactive Oxygen Species
SISBIO	Biodiversity Authorization and Information System
SOD	Superoxide Dismutase
TLR	Toll-Type Receiver
TLR2/TLR4	Toll-Type Receiver 2/Toll Type Receiver 4
TMB	3,3′,5,5′-Tetrametilbenzidina
ZI	Zymosan
ZI/HNS	Zymosan Opsonized with Human Serum
ZI/HIHS	Zymosan Treated with Heat-Inactivated Human Serum
